# Mutagenic assessment of chemotherapy and Smac mimetic drugs in cells with defective DNA damage response pathways

**DOI:** 10.1038/s41598-018-32517-9

**Published:** 2018-09-26

**Authors:** Mark A. Miles, Christine J. Hawkins

**Affiliations:** Department of Biochemistry and Genetics, La Trobe Institute for Molecular Science, La Trobe University, Victoria, Australia

## Abstract

DNA damaging therapies can spur the formation of therapy-related cancers, due to mis-repair of lesions they create in non-cancerous cells. This risk may be amplified in patients with impaired DNA damage responses. We disabled key DNA damage response pathways using genetic and pharmacological approaches, and assessed the impact of these deficiencies on the mutagenicity of chemotherapy drugs or the “Smac mimetic” GDC-0152, which kills tumor cells by targeting XIAP, cIAP1 and 2. Doxorubicin and cisplatin provoked mutations in more surviving cells deficient in ATM, p53 or the homologous recombination effector RAD51 than in wild type cells, but suppressing non-homologous end joining (NHEJ) by disabling DNA-PKcs prevented chemotherapy-induced mutagenesis. Vincristine-induced mutagenesis required p53 and DNA-PKcs but was not affected by ATM status, consistent with it provoking ATM-independent p53-mediated activation of caspases and CAD, which creates DNA lesions in surviving cells that could be mis-repaired by NHEJ. Encouragingly, GDC-0152 failed to stimulate mutations in cells with proficient or defective DNA damage response pathways. This study highlights the elevated oncogenic risk associated with treating DNA repair-deficient patients with genotoxic anti-cancer therapies, and suggests a potential advantage for Smac mimetic drugs over traditional therapies: a reduced risk of therapy-related cancers.

## Introduction

Anti-cancer drugs that inhibit topoisomerase-II proteins or cause DNA-adducts or interstrand crosslinks generate DNA double strand breaks (DSBs) that can be recognized by DNA damage response pathways. The accumulation of unrepaired DSBs can lead to apoptotic cell death, however cancer cells can develop ways to bypass cell death pathways leading to chemotherapy resistance^1^. Cells that evade DNA damage-induced apoptosis may acquire genomic alterations due to the mis-repair of DNA damage^2^. The direct effects of genotoxic drugs on non-cancerous cells may contribute to the formation of therapy-related second cancers, for example agents that alkylate DNA or target topoisomerase-II provoke chromosomal abnormalities that characterize therapy-related acute myeloid leukemia or myelodysplastic syndrome (t-AML/MDS)^3,4^. The incidence of second cancers has risen over the last three decades^5^; childhood cancer survivors have a six-fold increased risk of developing a subsequent neoplasm^6^. Risk factors for cancer survivors acquiring subsequent malignancies include exposure to DNA damaging therapies and variations in genes essential for maintaining genomic stability^7,8^. These risk factors may interact, rendering individuals with germline impairments in DNA damage responses especially sensitive to the oncogenic activity of genotoxic therapies^9,10^.

A number of proteins are crucial for detecting and responding to DNA damage. Upon recruitment of the Mre11-Rad50-Nbs1 (MRN) complex to sites of DSBs, ataxia-telangiectasia mutated (ATM) becomes activated and phosphorylates proteins including H2AX, checkpoint kinase 2 (Chk2) and the tumor suppressor p53, to promote cell cycle arrest, DNA repair or apoptosis^11–13^. P53 plays a central role in detecting and responding to cellular stresses such as oncogene activation, hypoxia and DNA damage^14^. Levels of p53 are suppressed by the E3 ubiquitin ligase MDM2 but stress signals activate post-translational modifications, including phosphorylation by ATM^15^, that stabilize p53, increasing its levels and allowing for transcription of target genes involved in apoptosis, cell cycle arrest, senescence and DNA repair^16^. Intrinsic apoptosis involves p53-mediated upregulation of pro-apoptotic Bcl-2 relatives to promote mitochondrial outer membrane permeabilization (MOMP) and caspase activation via the apoptosome^17^.

Mammalian cells can repair DSBs by two distinct pathways. Homologous recombination (HR) can accurately repair DNA damage when an intact template is available, whereas inaccurate repair can occur by non-homologous end-joining (NHEJ)^18^. DNA-PKcs is the catalytic subunit of the DNA-PK holoenzyme. It becomes active following association with the Ku70/Ku80 heterodimer bound to free DNA ends^19^, facilitating the re-ligation of DNA ends via NHEJ^20^. In contrast, HR utilizes a homologous template to repair DSBs with high fidelity. RAD51 is a key protein in this repair process, which interacts with proteins including RPA and RAD51 paralogs to achieve homology search and DNA strand invasion^21^.

Familial cancer predisposition syndromes, such as Li-Fraumeni and ataxia telangiectasia, can result from inherited mutations in genes, like p53 and ATM, that control responses to DNA damage^22^. Germline mutations in ATM and p53 were also commonly detected in patients with sporadic cancers, emphasizing the contribution of these genes to tumorigenesis^23–28^. A single nucleotide polymorphism of RAD51 has also been linked to an elevated risk of a number of cancer types, especially amongst Caucasians^29^. These correlations imply that oncogenesis may be facilitated through defects in DNA repair that promote genomic instability^30^. These defects have also been proposed to influence responses to certain traditional therapies^31,32^, and thus may render this sub-set of patients especially prone to develop therapy-related cancers following treatment with DNA-damaging chemotherapy or radiotherapy.

Members of the Inhibitor of Apoptosis (IAP) family of proteins are often over-expressed in human cancer^33^. “Smac mimetics”, also known as “IAP antagonists”, can eliminate cancer cells by blocking XIAP-mediated caspase inhibition, and/or degrading cIAP1 and 2 to divert TNF-R1 signaling away from pro-survival pathways towards apoptotic and/or necroptotic cell death^34^. IAP antagonists can sensitize cancer cells to chemotherapeutics and other targeted therapies^35–37^, potentially bypassing mechanisms of chemoresistance. Engaging necroptosis through these drugs or other activators is an emerging alternative to traditional anti-cancer therapies^38^. An additional advantage is that Smac mimetics do not induce DNA damage in order to initiate cell death, and seem to lack mutagenic activity^39^. A number of Smac mimetics have been successfully tested in pre-clinical studies and early phase clinical trials with potential combinational therapies with chemotherapy and/or other targeted therapies^40^.

This study compared the ability of genotoxic chemotherapy drugs and Smac mimetic treatment to mutate cells bearing genetic defects that prevent them from effectively responding to DNA damage. GDC-0152 is a well-tolerated Smac mimetic with high affinity for XIAP, cIAP1 and 2^41^. The mutagenic capacities of clinically-used chemotherapies and GDC-0152 were quantitated in wild type TK6 cells or derivatives engineered to lack signaling components required for DNA damage responses: ATM, p53, DNA-PKcs or RAD51. Mutations were assessed using the HPRT assay, which identifies the acquisition of loss-of-function HPRT mutations in clonogenically competent cells following drug treatment, as HPRT mutant cells can survive and proliferate in 6-thioguanine (6-TG)^42^.

## Results

### Loss of ATM function promotes mutagenesis by doxorubicin and cisplatin, but not vincristine or GDC-0152

Drugs that damage DNA by direct interaction with the DNA or topoisomerase enzymes, or indirectly via apoptotic nucleases, can provoke mutations in surviving cells^43^. Smac mimetics are a new class of anti-cancer drugs that provoked negligible mutagenesis in our initial characterization^39^, however it is not yet known whether these drugs maintain this lack of mutagenic activity in cells with DNA repair deficiencies. For this study, we compared the mutagenesis of the Smac mimetic GDC-0152 alongside commonly used chemotherapies. Sub-lethal doses of GDC-0152 failed to provoke any HPRT mutations in parental TK6 cells compared to an equivalently sub-lethal dose of doxorubicin (Supplementary Fig. 1), consistent with our previous observations using other Smac mimetics^39,44^.

**Figure 1:**
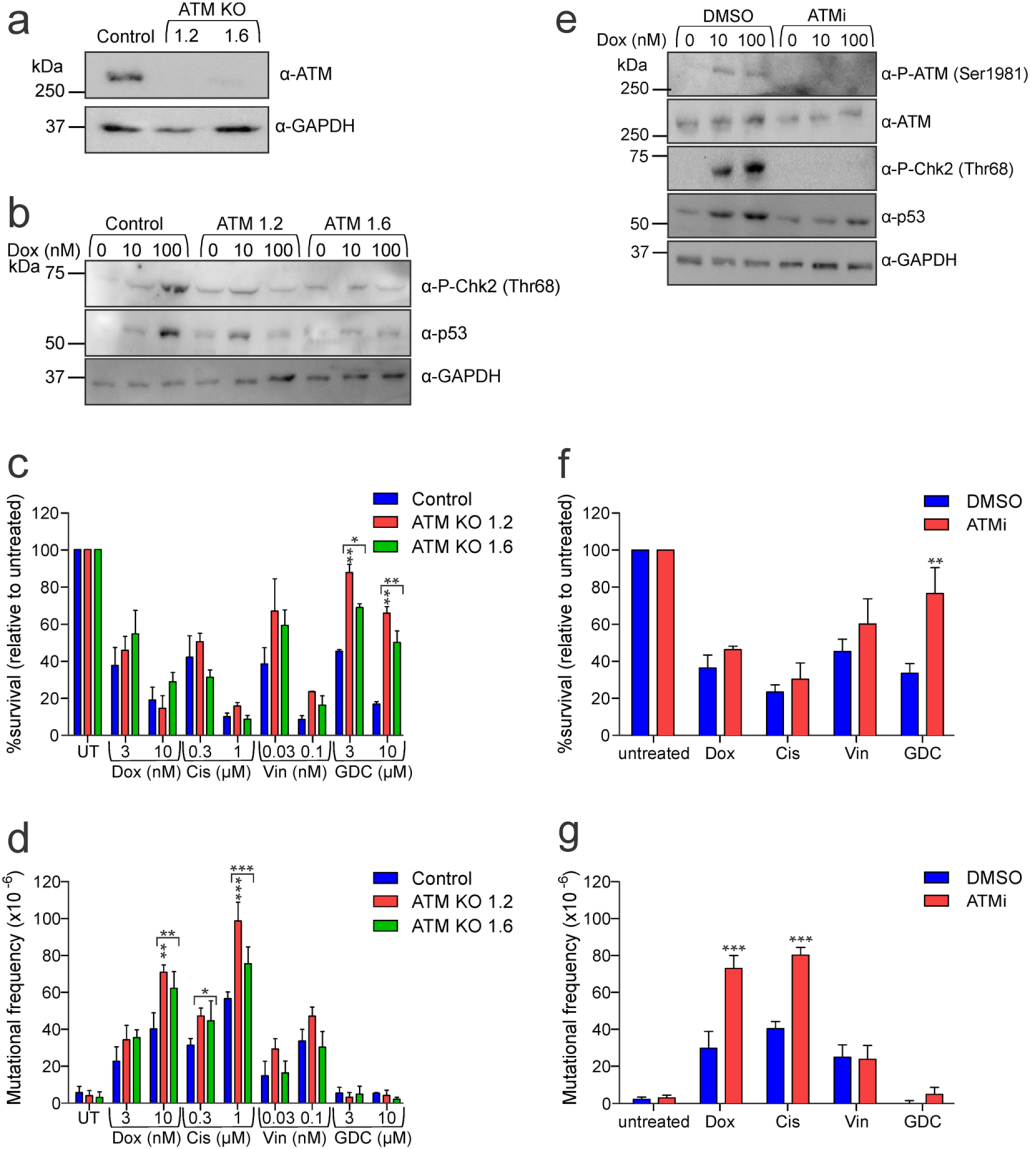
Lack of ATM activity enhances mutations induced by doxorubicin and cisplatin but does not render GDC-0152 mutagenic (**a**) ATM protein expression in ATM expressing (control) and ATM knockout cells was assessed by immunoblot. Probing for GAPDH was used as a loading control. (**b**) Cells were treated with doxorubicin for 5 hours then lysates were subjected to immunoblotting. (**c**) Cells were treated with indicated doses of drugs or left untreated (UT) for 24 hours, harvested and clonogenicity assays performed to determine the proportion of cells maintaining clonogenic competency. (**d**) Surviving cells were grown in 6-TG to select for the emergence of any HPRT mutants. (**e**) TK6 cells were pre-treated with 10 μM KU-60019 (ATMi) or DMSO for 1 hour, exposed to doxorubicin, then lysates were subjected to immunoblotting. KU-60019 pre-treated cells were exposed to 10 nM doxorubicin (Dox), 1 μM cisplatin (Cis), 0.1 nM vincristine (Vin), 10 μM GDC-0152 (GDC) or untreated for 24 hours. Cells were harvested and (**f**) clonogenicity or (**g**) HPRT assays were performed on surviving cells. Error bars represent mean ± SEM from at least three independent experiments. Two-way ANOVA analyses with Bonferroni post-tests were used to estimate the probability that random chance accounted for the differences observed in drug responses between control cells and either each ATM null line or both KO lines (denoted by the square brackets) (**c**,**d**), or DMSO-treated versus inhibitor-treated cells (**f**,**g**). P values < 0.05 are designated with asterisks: ***<0.001, **<0.01, *<0.05. (**a**,**b**,**e**) Full length images of the immunoblots are provided in Supplementary Fig. 5.

We first tested if the mutagenesis induced by chemotherapy drugs was exacerbated in cells lacking ATM activity. ATM knockout cells (KOs) were generated using CRISPR/Cas9 gene editing (Fig. 1a). Doxorubicin treatment induced Chk2 phosphorylation and p53 stabilization in parental cells but not in ATM-deficient clones (Fig. 1b), consistent with impaired ATM signaling in the gene-edited lines. Concentrations of doxorubicin, cisplatin, vincristine or GDC-0152 that impacted similarly on the clonogenic survival of the parental cells were then applied to the parental and knockout cells. ATM loss did not alter sensitivity to doxorubicin or cisplatin, but slightly reduced the cells’ sensitivity to vincristine and significantly enhanced clonogenicity following exposure to GDC-0152 (Fig. 1c). Doxorubicin and cisplatin provoked HPRT mutations in significantly more cells lacking ATM expression than control cells, as measured by quantitating the emergence of 6-TG-resistant clones, however vincristine-induced mutagenesis was unaffected by ATM status (Fig. 1d). GDC-0152 failed to trigger mutations even in cells lacking ATM (Fig. 1d).

To explore possible off-target effects of the CRISPR technique that we used to abolish ATM expression, or potential selective pressure during the generation and culturing of ATM knockout clones, we complemented the genetic ablation experiments by chemically inhibiting ATM with KU-60019 (ATMi)^45^. DNA damage induced by doxorubicin caused auto-phosphorylation of ATM at serine-1981 as expected^46^ but this was absent in ATMi pre-treated cells (Fig. 1e). Doxorubicin-treated cells lacked Chk2 phosphorylation and p53 stabilization when ATM was chemically inhibited, implying that ATM remained inactive in these cells. Inhibition of ATM, like its genetic inactivation, significantly enhanced clonogenic survival after GDC-0152 treatment (Fig. 1f). Mutation frequencies were significantly increased following doxorubicin or cisplatin exposure, but vincristine provoked similar levels of HPRT mutagenesis in the presence or absence of the ATM inhibitor. ATM inhibition neither boosted the basal frequency of HPRT mutations, nor provoked mutagenesis following exposure to GDC-0152 (Fig. 1g).

### Loss of p53 function promotes mutagenesis after direct DNA damage but reduces vincristine-induced mutagenesis; GDC-0152 is non-mutagenic regardless of p53 status

**Figure 2:**
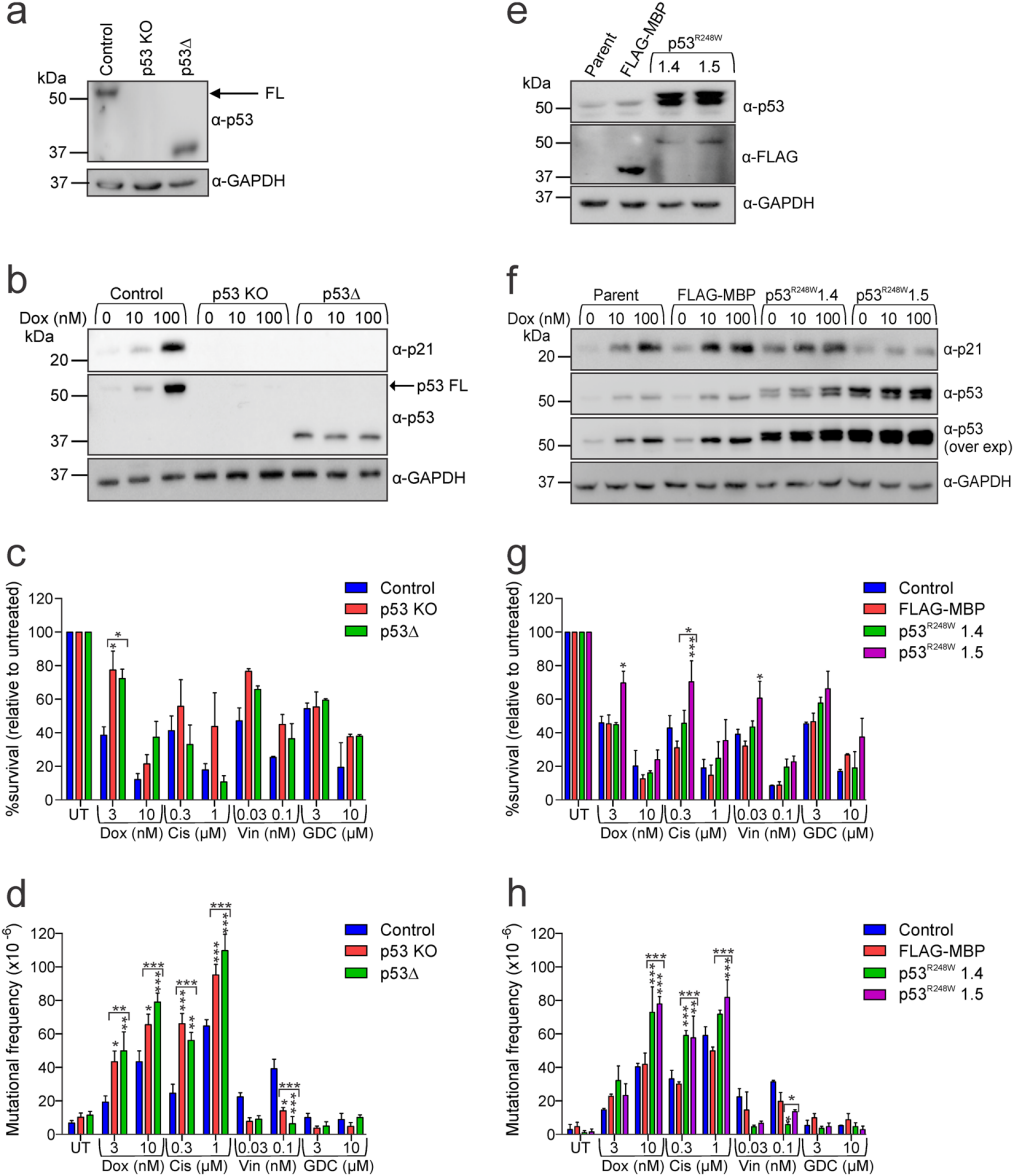
Lack of functional p53 enhances mutations induced by doxorubicin and cisplatin but reduces vincristine-mediated mutagenesis; GDC-0152 is non-mutagenic regardless of p53 status (**a**) p53 protein expression in p53 expressing cells (control), a p53 knockout line (KO) and a p53 internal deletion mutant line (Δ) was assessed by immunoblot. The blot was reprobed with GAPDH to indicate loading. (**b**) Cells were treated with doxorubicin for 5 hours then lysates were subjected to immunoblotting. (**c**) Cells were treated with the indicated doses of drugs or left untreated (UT) for 24 hours, harvested and clonogenicity assays performed to determine the proportion of cells maintaining clonogenic competency. (**d**) Surviving cells were grown in 6-TG to select for the emergence of any HPRT mutants. (**e**) p53, FLAG and GAPDH protein expression in parental cells and clones expressing FLAG-tagged constructs were assessed by immunoblot. (**f**) Cells were treated with doxorubicin for 5 hours then lysates were subjected to immunoblotting. Cells were then treated with indicated doses of drugs or left untreated (UT) for 24 hours then (**g**) clonogenicity and (**h**) HPRT assays performed on surviving cells. (**c**,**d**,**g**,**h**) Two-way ANOVA analyses with Bonferroni post-tests were used to estimate the probability that random chance accounted for the differences observed in drug responses between control cells and either each p53 mutant line, or both deleted or both mutant lines (denoted by the square brackets). P values < 0.05 are denoted with asterisks: ***<0.001, **<0.01, *<0.05. (**a**,**b**,**e**,**f**) Full length images of the immunoblots are provided in Supplementary Fig. 6.

P53 is frequently mutated in human cancer and is crucial for the regulation of apoptosis and cell cycle arrest, especially in response to DNA damage^47^. Parental TK6 cells were reported to express wild type p53^48^. CRISPR/Cas9 gene editing generated a line lacking p53 and another clone expressing a p53 protein containing an internal deletion (p53Δ) (Fig. 2a and Supplementary Fig. 2). These lines were treated with doxorubicin to assess their DNA damage responsiveness. Both clones exhibited null phenotypes: neither exhibited induction of p21 expression following doxorubicin treatment, unlike wild type cells (Fig. 2b). Basal levels of p53 in the p53Δ clone were higher than in the parental cells, but were not further boosted by doxorubicin treatment (Fig. 2b), consistent with this variant lacking residues required for MDM2 binding (and hence proteasomal degradation)^49^. Treatment with the lower dose of doxorubicin was significantly less toxic in the absence of functional p53, confirming the involvement of p53-mediated apoptotic signaling (Fig. 2c). P53-deficiency seemed to exert a mild protective effect following vincristine treatment, although this was not statistically significant. Interestingly, doxorubicin and cisplatin generated substantially more frequent HPRT mutations in the p53 mutant lines than in wild type cells, while fewer cells lacking p53 acquired mutations after vincristine exposure (Fig. 2d). Very few HPRT mutant clones emerged in untreated and GDC-0152-treated cells even when p53 function was absent.

Missense mutations, often located within the DNA binding domain, are the most common p53 alterations in tumors^50^. To model this type of loss-of-function variant, we generated TK6 stable lines expressing a FLAG-tagged p53 protein containing the common R248W mutation (p53^R248W^) or Maltose Binding Protein (FLAG-MBP) as a control (Fig. 2e and Supplementary Fig. 2). P53^R248W^ mutant clone 1.5 lacked p21 induction upon doxorubicin treatment, indicating a severe loss-of-function phenotype (Fig. 2f). These cells were significantly less sensitive to low concentrations of chemotherapy drugs than cells only expressing wild type p53 (Fig. 2g). The other p53 mutant clone (1.4) seemingly harbored residual p53 function, as doxorubicin treatment led to somewhat elevated p21 and p53 levels, although the magnitude of the response was more muted than that observed in parental or MBP-expressing cells (Fig. 2f). This partial loss of p53 activity did not significantly affect clonogenic survival following drug treatment (Fig. 2g). Doxorubicin and cisplatin provoked mutations more frequently in both clones expressing p53^R248W^ than the control cells (Fig. 2h). Vincristine was less mutagenic to cells expressing p53^R248W^ than wild type p53. GDC-0152 did not induce HPRT mutations regardless of p53 status (Fig. 2h).

**Figure 3:**
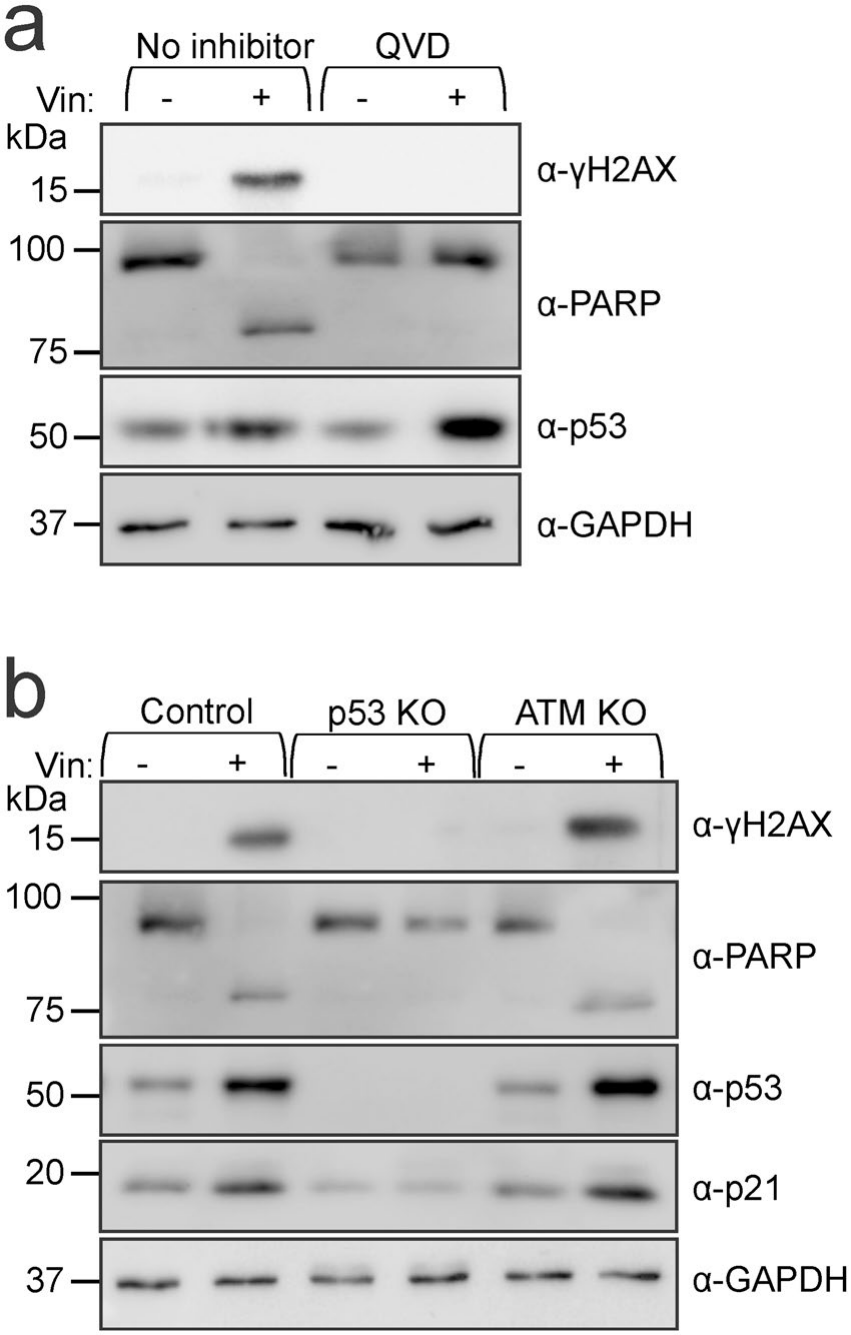
DNA damage induced by vincristine is p53-dependent (**a**) TK6 cells were incubated with no inhibitor or 10 μM Q-VD-OPh (QVD) for 1 hour then treated with or without 1 nM vincristine for 16 hours. (**b**) Control, p53 KO and ATM KO (1.2) lines were also treated with or without 1 nM vincristine for 16 hours. All cells were processed for immunoblotting. Full length images of the blots are provided in Supplementary Fig. 7.

These data revealed that mutations arose more frequently following doxorubicin and cisplatin exposure in the absence of ATM or p53. In contrast, lack of functional ATM had no effect on vincristine-mediated mutagenesis, and acquisition of those mutations was p53-dependent. Vincristine-induced mutagenesis was previously noted to also require executioner caspases and the apoptotic nuclease CAD^43^. Vincristine stabilized p53, induced PARP cleavage and provoked DNA damage, as indicated by phosphorylation of H2AX (γH2AX) (Fig. 3a). Each of these responses except p53 stabilization were prevented by pre-treatment with the pan-caspase inhibitor QVD, implying that caspases became activated after vincristine-mediated p53 stabilization. Vincristine treatment provoked PARP cleavage and H2AX phosphorylation in control and ATM knockout cells but not p53 null cells (Fig. 3b). These observations reveal that activated caspases and DNA damage occur following vincristine-mediated p53 stabilization and subsequent pro-apoptotic signaling, and that phosphorylation of H2AX and p53 following exposure to vincristine is not performed by ATM.

### Non-homologous end joining DNA mis-repair is responsible for chemotherapy-induced mutagenesis

**Figure 4:**
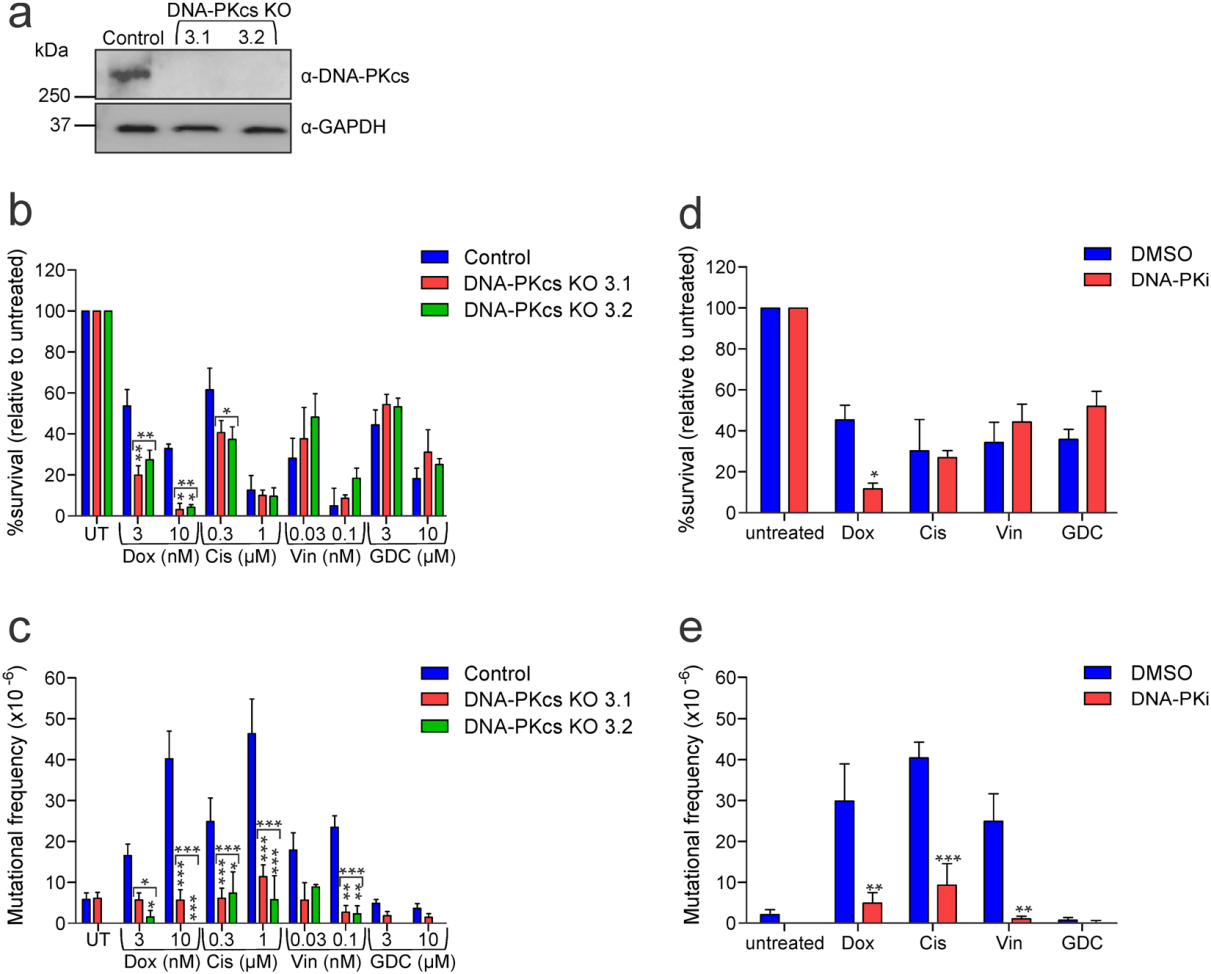
Lack of functional DNA-PKcs reduces chemotherapy-induced mutations (**a**) DNA-PKcs protein expression in DNA-PKcs expressing (control) and DNA-PKcs knockout cells was assessed by immunoblot. Probing for GAPDH was used as a loading control. (**b**) Cells were treated with indicated doses of drugs or left untreated (UT) for 24 hours, harvested and clonogenicity assays were performed to determine the proportion of cells maintaining clonogenic competency. (**c**) Surviving cells were grown in 6-TG to select for the emergence of any HPRT mutants. (**d**) TK6 cells were pre-treated with 10 μM KU-57788 (DNA-PKi) or DMSO for 1 hour, then cultured in media containing 10 nM doxorubicin (Dox), 1 μM cisplatin (Cis), 0.1 nM vincristine (Vin), 10 μM GDC-0152 (GDC) or untreated for 24 hours. Cells were harvested and clonogenicity or (**e**) HPRT assays were performed on surviving cells. Error bars represent mean ± SEM from at least three independent experiments. Two-way ANOVA analyses with Bonferroni post-tests were used to estimate the probability that random chance accounted for the differences observed in drug responses between control cells and either each DNA-PKcs null line or both KO lines (denoted by the square brackets) (**b**,**c**), or DMSO-treated versus inhibitor-treated cells (**d**,**e**). P values < 0.05 are denoted with asterisks: ***<0.001, **<0.01, *<0.05. (**a**) Full length images of the immunoblots are provided in Supplementary Fig. 7.

The data presented above indicate that cells lacking crucial DNA damage response proteins are more sensitive to the mutagenic effects of DNA damaging anti-cancer drugs. We reasoned that these mutations are most likely to arise due to inaccurate DNA repair, probably due to low fidelity NHEJ^20^. To investigate the contribution of NHEJ to mutagenesis stimulated by anti-cancer drugs, we generated cell lines lacking expression of DNA-PKcs (Fig. 4a), which is an essential kinase involved in NHEJ^51^. Clonogenic survival was severely reduced in cells lacking DNA-PKcs expression after doxorubicin exposure, and less markedly after cisplatin treatment (Fig. 4b). Strikingly, loss of DNA-PKcs abolished the mutagenic activity of the chemotherapy drugs (Fig. 4c). Cells treated with GDC-0152 showed only background mutation frequencies, regardless of DNA-PKcs status.

These assays were repeated in cells pre-treated with KU-57788 (DNA-PKi), a DNA-PKcs inhibitor^52^. Doxorubicin caused dramatic clonogenic death in the cells lacking DNA-PKcs activity, but inhibition of this kinase did not have a major effect on the sensitivity of the cells to the other drugs (Fig. 4d). The frequencies of HPRT mutations stimulated by each chemotherapy drug were significantly reduced when DNA-PKcs was inhibited (Fig. 4e). GDC-0152 was not mutagenic, whether DNA-PKcs was inhibited or not.

**Figure 5:**
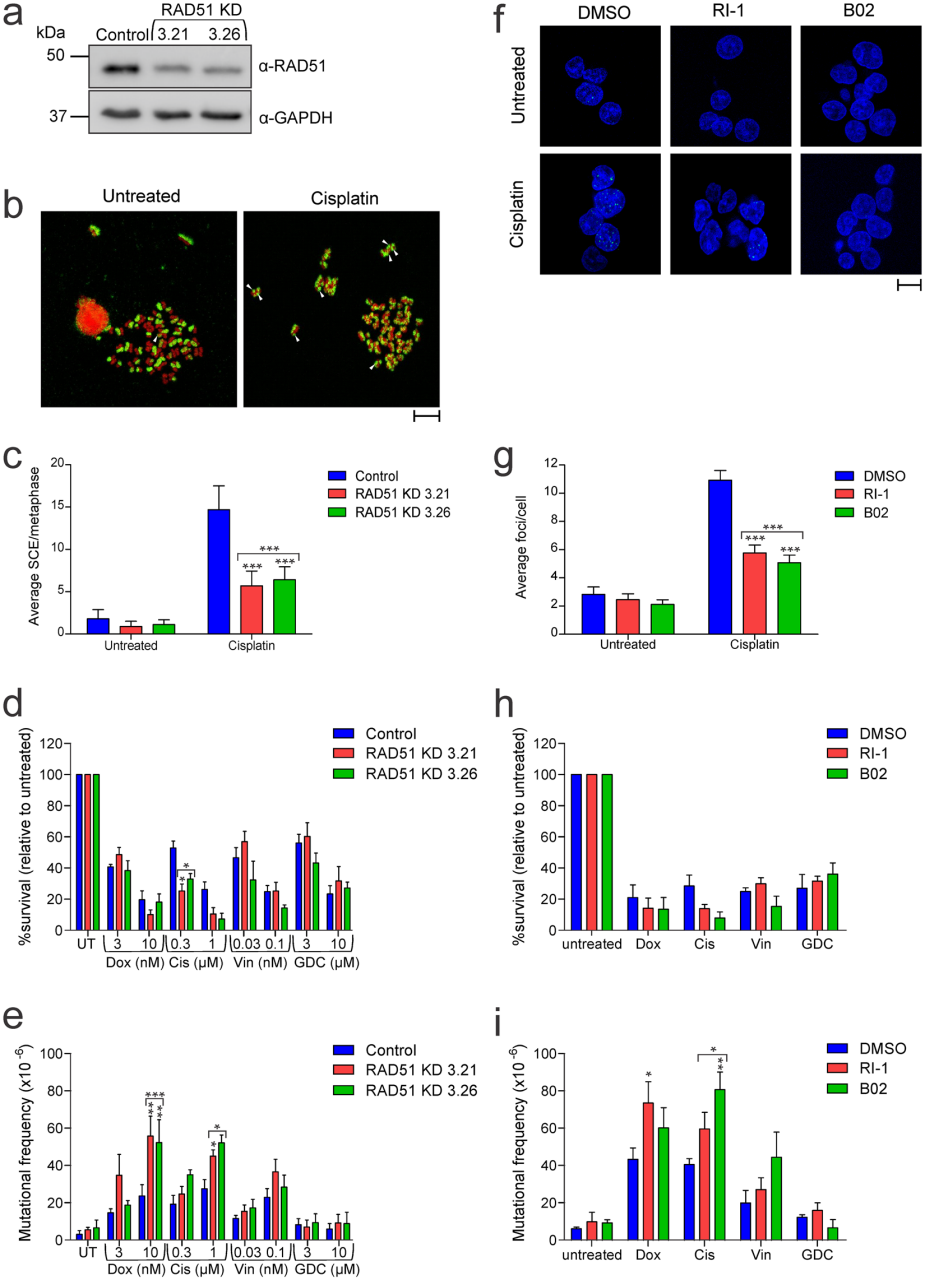
Limited RAD51 activity enhances mutations induced by doxorubicin, cisplatin and vincristine but does not render GDC-0152 mutagenic (**a**) RAD51 protein expression in RAD51 expressing (control) and RAD51 knockdown (KD) cells was assessed by immunoblotting. Blots were reprobed with an antibody to GAPDH to indicate loading. The blots shown are representative of three independent replicates. (**b**) Cells were grown in BrdU for 24 hours and treated with 1 μM cisplatin for a further 24 hours. Fixed cells were spotted onto microscope slides, chromosomes stained with anti-BrdU primary and FITC conjugated secondary antibodies and counterstained with PI to visualize DNA content. Representative images of metaphase spreads are shown. White markers indicate some sister chromatid exchange (SCE) events. Scale bar is 10 μm. (**c**) The number of SCE events were counted from 30 metaphase spreads from each cell line and each treatment. (**d**) Cells were treated with the indicated doses of drugs or left untreated (UT) for 24 hours, harvested and clonogenicity assays were performed to determine the proportion of cells maintaining clonogenic competency. (**e**) Surviving cells were grown in 6-TG to select for the emergence of any HPRT mutants. (**f**) TK6 cells were pre-treated with 30 μM RI-1, 20 μM B02 or DMSO for 1 hour then exposed to 1 μM cisplatin for 24 hours. Fixed cells were stained with an anti-RAD51 primary antibody and FITC conjugated secondary antibody then counterstained with DAPI. Representative immunofluorescent images are shown. Scale bar is 10 μm (**g**) The number of RAD51 foci per nucleus were counted from at least 50 nuclei for each treatment. Following DMSO, RI-1 or B02 pre-treatment, cells were incubated in normal media or with 10 nM doxorubicin (Dox), 1 μM cisplatin (Cis), 0.1 nM vincristine (Vin) or 10 μM GDC-0152 (GDC) for 24 hours then harvested. (**h**) Clonogenicity assays were performed while (i) surviving cells were grown in 6-TG to select for the emergence of any HPRT mutants. (**c**–**e**, **g**–**i**) Error bars represent mean ± SEM from three independent experiments. Two-way ANOVA analyses with Bonferroni post-tests were used to estimate the probability that random chance accounted for the differences observed in drug responses between control cells and either each RAD51 KD line or both KD lines denoted by the square brackets) (**c**–**e**), or DMSO-treated versus inhibitor-treated cells (**g**–**i**). P values < 0.05 are denoted with asterisks: *** < 0.001, ** < 0.01, * < 0.05. (**a**) Full length images of the immunoblots are provided in Supplementary Fig. 7.

HR would be likely to mend double strand breaks during S/G2 phases of the cell cycle. To model a context in which these cells instead rely on NHEJ for repair, we targeted RAD51, which facilitates HR. CRISPR/Cas9 gene targeting failed to generate any homozygous RAD51 mutants but “knockdown” (KD) clones were obtained, which expressed reduced levels of RAD51 (Fig. 5a and Supplementary Fig. 3). Sister chromatid exchanges (SCE) are mediated by HR and can be stimulated by DNA damage during replication^53^ (Fig. 5b). RAD51 knockdown cells exhibited significantly fewer SCE events following exposure to cisplatin compared to controls (Fig. 5c) confirming compromised HR activity in these cells. These knockdown clones were more sensitive than wild type cells to clonogenic death induced by cisplatin but similarly sensitive to doxorubicin, vincristine and GDC-0152 (Fig. 5d). Mutation frequencies for doxorubicin and cisplatin were higher in cells with reduced RAD51 levels (Fig. 5e), but vincristine-induced mutations occurred with similar frequencies in RAD51 knockdown and wild type cells. GDC-0152 treatment yielded only background mutation frequencies in all cell lines. To validate the RAD51 knockdown data, we pre-treated cells with two different selective chemical inhibitors of RAD51, RI-1 and B02^54,55^. Immunofluorescent detection of RAD51 nuclear foci can be detected upon DNA damage as a measure of RAD51 activity^56^. Cells pre-treated with either RAD51 inhibitor contained fewer foci upon cisplatin treatment when compared to DMSO control (Fig. 5f,g) implying RAD51 was less active in these cells. The RAD51 inhibitors slightly reduced clonogenicity following cisplatin treatments, but not significantly, and sensitivity to the other drugs remained unchanged (Fig. 5h). These pharmacological RAD51 inhibitors amplified the mutagenic effects of doxorubicin or cisplatin (Fig. 5i).

## Discussion

DNA damaging chemotherapies have improved cancer survival rates^57^, however some cancers fail to respond to such treatments, often due to enhanced intrinsic pro-survival properties and/or defects in the DNA damage response. These drugs can also contribute to the formation of therapy-related second cancers in survivors, which may be augmented by a genetic predisposition, especially defects in genes that help faithfully fix DNA lesions^7^. This study sought to define the impact of defective DNA damage responses on mutagenicity provoked by chemotherapy drugs that cause double stranded DNA breaks, to model the likely oncogenic potential of these therapies upon cancer patients with germline mutations that impair DNA repair processes. Smac mimetics are a promising class of drugs that may overcome traditional blocks in apoptotic pathways commonly activated by chemotherapy. The mutagenicity of the Smac mimetic GDC-0152 was compared with currently used chemotherapeutics, in cells with proficient or deficient responses to DNA damage.

Genetic ablation or chemical inhibition demonstrated that lack of functional ATM, a key DNA damage sensor and transducer of the DNA damage response, enhanced mutagenesis induced by doxorubicin (a topoisomerase-II inhibitor) and cisplatin (a platinating agent), in accordance with previous reports that ATM inhibition reduced levels of ɣH2AX following treatment with these drugs^58,59^. Similarly, mutagenesis by these agents was also enhanced following CRISPR/Cas9 gene editing of the ATM substrate p53, an important regulator of cell cycle arrest, senescence and apoptosis, or over expression of a cancer-associated p53 mutant. The increase in clonogenic survival triggered by the lower dose of doxorubicin in p53 defective cells presumably reflects its stimulation of p53-dependent intrinsic apoptotic signaling, via transcriptional upregulation of the pro-apoptotic BH3-only proteins Puma and Noxa^60^, and senescence via induction of p53 target genes including p21 and PML^61^. Some of our data suggest that low dose cisplatin may also provoke a similar p53-dependent cell death and/or senescence pathway, but the heterogeneity in the responses of p53 knockout and mutant cells to cisplatin preclude definitive conclusions regarding this. In contrast to p53 inhibition/deficiency, ATM loss or inhibition failed to significantly enhance clonogenic survival after exposure to the lower concentration of doxorubicin. These data contrasted with a prior report documenting that ATM inhibition subtly exacerbated doxorubicin toxicity in prostate cancer cells^58^, and was somewhat surprising, as ATM often activates p53 in doxorubicin-treated cells, either directly or via Chk2 activation^62^. However, other stress kinases, like ATR, can also phosphorylate p53^63,64^ so it is possible that these enzymes may substitute for ATM in promoting p53-dependent loss of clonogenicity in this context. The clonogenic survival of cells exposed to higher doxorubicin concentrations, or cisplatin, was not significantly enhanced by loss of p53 or ATM. We infer that most cells exposed to high dose doxorubicin or cisplatin lost the ability to survive and proliferate due to widespread genomic fragmentation, which exceeded the cell’s repair capacity, and/or perhaps MK2-mediated mitotic catastrophe, a p53-independent process that can be triggered by these agents^65^. P53 deficiency had a much more pronounced effect on mutagenicity than clonogenicity, reinforcing the importance of its roles in DNA repair^66,67^ in addition to preventing the survival or proliferation of cells bearing widespread genomic damage. We previously attributed a proportion of doxorubicin-induced mutations to caspase-mediated activation of the nuclease CAD^43^, however this study revealed that loss of ATM or p53 activity enhanced net mutagenesis by doxorubicin, implying the anti-mutagenic roles of ATM and p53 (such as homologous recombination and senescence) outweigh their ability to stimulate CAD-dependent mutagenesis via sub-lethal apoptotic signaling.

We also defined the type of DNA repair pathways activated in cells in response to chemotherapy-induced DNA damage. Suppression of NHEJ by knocking out DNA-PKcs or inhibiting its activity reduced the clonogenic survival of doxorubicin- or cisplatin-treated cells (as had been previously reported for irradiated cells^68^), and also largely abolished HPRT mutations amongst cells that survived these treatments. These data imply that DNA-PKcs-deficient cells harboring chemotherapy-induced DNA damage either died or achieved faithful DNA repair via accurate HR. Consistent with this, suppression of HR by downregulating RAD51 or inhibiting its activity exacerbated the ability of doxorubicin and cisplatin to provoke mutations in surviving cells. Although RAD51 enabled cells to accurately repair DNA damage caused by both drugs, RAD51-deficient cells bearing cisplatin-induced lesions were more likely than those bearing doxorubicin-induced damage to forfeit clonogenicity, consistent with the previously noted importance of HR in suppressing cisplatin-induced toxicity^69^. The mutagenicity data demonstrate that cells which survived either treatment mended their DNA via error-prone mechanisms requiring DNA-PKcs. To our knowledge, this is the first study to directly measure the impact of ATM, p53, DNA-PKcs or RAD51 status on the ability of doxorubicin or cisplatin to stimulate mutations in clonogenically-competent cells, thus modelling events that could lead to therapy-related cancers *in vivo*. However, researchers have previously documented increased spontaneous and radiation-induced mutagenesis when p53^70–75^, ATM^76,77^ or RAD51^78^ activity were attenuated or lost, and lower mutation rates when NHEJ was compromised^79,80^. Inhibition of ATM or DNA-PK were previously documented to reduce H2AX phosphorylation following treatment with cisplatin^59^, whereas doxorubicin-mediated γH2AX was lessened by ATMi treatment but augmented by DNA-PKi treatment^58^. Although we did not monitor γH2AX following doxorubicin or cisplatin treatment, it probably was present in cells containing active ATM (and possibly DNA-PKcs), as H2AX is a well-established substrate of these kinases and γH2AX was detected in vincristine-treated TK6 cells. Our findings emphasize that, while H2AX phosphorylation provides useful information about cellular and treatment conditions that cause DNA breaks, it cannot be used as a surrogate marker of mutagenesis, as it marks lesions that may ultimately be repaired accurately or inaccurately^81^, or may remain unmended if the cell dies. In our experiments, ATM (or p53) loss increased the frequency with which doxorubicin or cisplatin provoked HPRT mutations, highlighting the dominant anti-mutagenic roles of these proteins, while DNA-PKcs deficiency blocked mutagenesis caused by these drugs, consistent with its role in mutation-prone DNA repair.

Mutagenesis by vincristine (a microtubule destabilizer) was unaffected by ATM status, but was p53-dependent. Reduction in p53 (or ATM activity) also enhanced clonogenic survival of vincristine-treated cells somewhat, although these differences were not statistically significant. Prior studies have reached differing conclusions regarding the importance of p53 for the cell death triggered by microtubule disrupting agents^82–86^, but to our knowledge this study provides the first indication of a role for p53 in mutagenesis stimulated such drugs. Intrinsic apoptotic signaling following improper chromosomal segregation is often regulated by p53^87^. We found that p53 loss prevented vincristine-induced H2AX phosphorylation, PARP cleavage and mutagenesis. The observation that p53 deficiency reduced vincristine-induced mutagenesis and H2AX phosphorylation, but ATM loss did not, suggests that activation of p53 (and the ensuing caspase-dependent DNA damage and mutations) is not solely accomplished by ATM under these conditions, consistent with previous reports linking ATR to p53 activation when mitosis is perturbed^88^. Suppressing NHEJ, through genetic or pharmacological targeting of DNA-PKcs, significantly reduced the frequency of HPRT mutations in vincristine-treated cells, but reducing homologous recombination failed to significantly impinge on vincristine-induced death or mutagenesis. These results, coupled with our previous observation that vincristine-mediated mutagenesis required executioner caspases and CAD^43^, suggest that mutagenesis following mitotic stress such as that provoked by vincristine results from activation of p53, probably by ATR, sublethal intrinsic apoptotic signaling including caspase-3/7 activity and the subsequent activation of CAD, and NHEJ-mediated mis-repair of CAD-mediated DNA damage. This model is consistent with previous reports that implicated NHEJ in the mis-repair of CAD-mediated DSBs^89^. Our data emphasize the mutagenic potential of NHEJ in response to DNA damage provoked by all three chemotherapy drugs tested.

Sensitivity to GDC-0152 remained similar among most conditions, although cells lacking ATM protein or kinase activity were less sensitive. TK6 cells neither stimulated autocrine TNFα production nor required the addition of recombinant TNFα to be sensitized to GDC-0152 killing, ruling out the possibility that depletion of ATM impacts TNFα-induced death as a result of GDC-0152 treatment (Supplementary Figs 1, 4). A connection between the action of IAP proteins and ATM has been previously documented^90^. Smac mimetics provoke cell death by preventing the ubiquitination of RIPK1, which facilitates its inclusion into complexes that stimulate cell death. One such complex, the cytosolic “ripoptosome”, can form without death receptor ligation and can provoke caspase-dependent apoptosis or caspase-independent necroptosis^90–92^, which has been reported to be regulated by both ATM and IAP proteins. ATM was observed to enhance ripoptosome formation, most potently in cells experiencing DNA damage, by promoting an association between Nemo and RIPK1, resulting in RIPK1 autophosphorylation and recruitment of FADD and caspase-8^90^. In that context, suppression of RIPK1 ubiquitination via treatment with a Smac mimetic sensitized cells to ATM-dependent ripoptotic death^90^. Although Biton and Ashkenazi (2011) focused on the activation of this pathway in the context of DNA damage, they noted that IAP antagonism also triggered detectable ripoptosome formation in the absence of DNA damaging treatments. We speculate that ATM is required to efficiently propagate ripoptosome activity in the TK6 cells used in this study, which is why its genetic or pharmacological removal dampened the toxicity provoked by GDC-0152-mediated IAP antagonism.

We were encouraged to observe that Smac mimetic treatment with GDC-0152 was non-mutagenic even in cells lacking ATM, p53 or RAD51, despite these three alterations exacerbating mutagenicity provoked by doxorubicin or cisplatin, and loss of ATM or RAD51 also augmenting vincristine-mediated mutagenicity. We suspect this to be the case for other monovalent and bivalent Smac mimetics, as we have previously shown drugs representing both subclasses to be non-mutagenic in TK6, LN18 and MEF cells^39,93^. We previously showed that sublethal executioner caspase activity, such as that stimulated by death receptor agonists, triggered mutations via activating the apoptotic nuclease CAD^43,94^. We speculate that the inability of sublethal exposure to Smac mimetics to elicit mutagenesis (via caspases and CAD, or any other mechanism) may stem from the ability of these drugs to stimulate caspase-independent necroptotic cell death; further work will be needed to explore this hypothesis.

We hope the lack of mutagenic activity we have observed following IAP antagonism *in vitro* will translate to a lack of mutagenic and oncogenic activity *in vivo*. It will be important to explore this, as *in vitro* systems cannot recapitulate some of the indirect effects of Smac mimetics that can manifest in treated animals or patients, including inflammatory cytokine production and resultant immunostimulation, which have been shown contribute to the anti-cancer effects of these drugs^95,96^. If the lack of mutagenic activity observed in this study is confirmed *in vivo*, cancer patients successfully treated with Smac mimetics may be less likely than those administered genotoxic therapies like doxorubicin or cisplatin to acquire therapy-related cancers. Cells bearing defects in ATM, p53 or RAD51 were particularly sensitive to the mutagenicity provoked by traditional anti-cancer drugs, implying that non-mutagenic therapies like Smac mimetics would be particularly beneficial for patients with germline mutations in DNA damage response genes.

## Materials and Methods

### Cell lines and reagents

TK6 cells and SKOV-3 cells were grown in RPMI-1640 containing HEPES buffer (Invitrogen; Carlsbad, CA, USA) supplemented with 10% heat inactivated FBS (Scientifix Life, Cheltenham, VIC, Australia). HEK-293T and LN18 cells were cultured in Dulbecco’s modified Eagle medium with high glucose (Invitrogen) supplemented with 10% heat inactivated FBS. All cells were grown at 37 °C in air supplemented with 5% CO_2_.

Drugs used were doxorubicin (Sigma; St. Louis, MO, USA), cisplatin (Sigma), vincristine (Selleck Chemicals; Houston, TX, USA), GDC-0152 (Selleck Chemicals), KU-60019 (Selleck Chemicals), KU-57788 (Selleck Chemicals), RI-1 (Selleck Chemicals), B02 (Selleck Chemicals) and 6-thioguanine (6-TG) (Sigma).

The following antibodies were used: rabbit anti-ATM (R2E2) (Cell Signaling Technology; Danvers, MA, USA), rabbit anti-P-ATM (Ser1981) (Cell Signaling), rabbit anti-DNA-PKcs (Santa Cruz Biotechnology; Dallas, TX, USA), rabbit anti-p53 (DO-1) (Santa Cruz), mouse anti-FLAG (M2) (Sigma), rabbit anti-p21 (Cell Signaling), mouse anti-PARP (46D11) (Cell Signaling), rabbit anti-phospho-H2AX (Ser 139) clone 20E3 (Cell Signaling), rabbit anti-P-Chk2 (Thr68) (Cell Signaling), mouse anti-GAPDH (Merck Millipore), mouse anti-TNFα (R&D Systems; Minneapolis, NM, USA), mouse anti-BrdU (BD Biosciences; San Jose, CA, USA), goat anti-rabbit-FITC (Merck Millipore), anti-mouse-FITC (Merck Millipore), donkey anti-rabbit-HRP (GE Healthcare Life Sciences; NJ, USA), and rabbit anti-mouse-HRP (Sigma).

### Immunoblotting

Immunoblotting was conducted as previously described^43^. Cells were lysed in RIPA lysis buffer (150 mM sodium chloride, 1.0% Triton X-100, 0.5% sodium deoxycholate, 0.1% SDS, 50 mM Tris, pH 8.0) supplemented with protease inhibitor cocktail (Roche; Basel, Switzerland), by pipetting and lysates cleared by centrifuging for 15 min at 16,100 g at 4 °C. Fifty micrograms of protein, as determined using the bicinchoninic acid (BCA) method (Micro BCA Protein assay kit, Thermo Fisher Scientific; IL, USA), was loaded on Tris-glycine gels, separated by sodium dodecyl sulfate-polyacrylamide gel electrophoresis and transferred onto Hybond PVDF 0.22 μm membrane (Millenium Science; Victoria, Australia). Membranes were blocked with 1% blocking reagent (Roche) in PBS, incubated with primary then secondary antibodies, then imaged following the addition of SuperSignal West Dura extended duration substrate (Thermo Fisher Scientific). Band intensity was quantitated using ImageJ (National Institute of Mental Health; Bethesda, MA, USA) software.

### Survival assays

Five-hundred thousand cells per ml were incubated in media alone or media containing drug, harvested, washed in PBS and counted. Cells were then seeded at an appropriate density (starting at 5 cells per well) in media containing 15% FCS and dispensed at 100 μl per well in 96-well round bottom plates. After 12–14 days, plates were scored for the number of wells with growth, and cloning efficiency (CE) calculated using the formula: CE = −ln (proportion of wells lacking growth)/ number of cells seeded per well. Annexin-V staining, propidium iodide uptake and CellTiter-Glo 2.0 assays were conducted as previously described^44^. For CellTiter-Glo 2.0, 10,000 TK6 cells, or 1000 SKOV-3 or LN18 cells were seeded per well of white 96-well plates in media alone or media containing drugs to a final volume of 75 μl. After treatment, CellTiter-Glo solution was mixed into each well, plates incubated for 10 min at room temperature, then luminescence recorded using a Spectromax M5 (Molecular Devices; Sunnyvale, CA, USA).

### HPRT mutation assay

HPRT assays were conducted according to a previously published method^39^. Briefly, untreated or treated cells (5 × 10^5^ cells/ml) were harvested, washed with PBS and counted for determining clonogenic survival. Remaining cells were then cultured in drug-free media for a 6 day lag period to ensure removal of any remaining endogenous HPRT activity in HPRT mutant cells. Cells were then seeded at 2 × 10^4^ cells per well in media containing 30 μM 6-TG and dispensed at 100 μl per well in 96-well round bottom plates. Cells were also seeded at 5 cells per well in drug-free media to determine plating efficiency (PE). Mutation frequency was calculated using the formula: MF = cloning efficiency (in 6-TG containing media)/plating efficiency (non-selective condition).

### Plasmids

A plasmid bearing the p53 R248W mutation (pCMV-Neo-Bam p53 R248W) was purchased from Addgene (Cambridge, MA, USA). The open reading frame was cloned into the pEF vector^97^ along with a FLAG epitope. The pEF-FLAG-MBP plasmid was previously described^43^. The following oligonucleotide pairs were annealed and ligated into BsmB1-cut pFgh1tUTG^98^.

ATM exon 2: 5′-TCCCTTGTTTCAGGATCTCGAATC-3′, 5′-AAACGATTCGAGATCCTGAAACAA-3′

TP53 exon 1: 5′-TCCCTCGACGCTAGGATCTGACTG-3′, 5′-AAACCAGTCAGATCCTAGCGTCGA-3′

PRKDC exon 1: 5′-TCCCGCCGGTCATCAACTGATCCG-3′, 5′-AAACCGGATCAGTTGATGACCGGC-3′

RAD51A exon 2: 5′-TCCCTAGCTCCTTCTTTGGCGCAT-3′, 5′-AAACATGCGCCAAAGAAGGAGCTA-3′

### Generation of CRISPR knockouts

The CRISPR/Cas9 doxycycline-inducible sgRNA lentiviral vector system^98^ was used as previously described^43^.

### Generation of mutant p53^R248W^ stable lines

Stable transfection of TK6 cells was accomplished by nucleofection utilizing the SF Nucleofector kit (Lonza, Allendale, NJ, USA)^43^.

### PCR and sequencing

Genomic PCR was conducted as previously described^99^. PCR products were sequenced via Sanger sequencing (AGRF; North Melbourne, VIC, Australia). The following primers were used:

RAD51: 5′-CTTCAAGCACCTCTGTGAAG-3′, 5′-CAGGTAACAGTCACCCCTGG-3′

p53: 5′-GTGCTTTCCACGACGGTGAC-3′, 5′-GAGAGATGGGGGTGGGAGGC-3′

### Immunofluorescence staining for RAD51 foci

Five hundred thousand cells were pre-treated with DMSO or inhibitor for 1 hour then incubated in 1 μM cisplatin or media for 24 hours. Cells were harvested, washed with PBS and fixed in formalin for 15 min at room temperature. Cells were washed again with PBS then permeabilized with 0.1% Triton X-100 in PBS for 15 min on ice. Cells were incubated for 1 hour in PBS containing 1% blocking reagent (Roche), then incubated with anti-RAD51 antibody (1:100) overnight at 4 °C. Cells were washed in PBS then incubated in anti-rabbit FITC antibody (1:200) for 1 hour in the dark. After washing again in PBS, cells were spotted onto a microscope slide in Antifade Mounting Medium with DAPI (Vector Laboratories, Burlingame, CA, USA) and analyzed on a Zeiss Confocal LSM 780 microscope (Carl Zeiss Pty Ltd, North Ryde, NSW, Australia).

### Sister chromatid exchange assay

This assay was based on a published method^100^. Two hundred thousand cells were incubated in 10 μM BrdU (Sigma) for 24 hours then exposed to 1 μM cisplatin for a further 24 hours. Colcemid (0.1 μg/ml) (Sigma) was then added to cells for the final 2 hours. After harvesting, cells were incubated in 75 mM KCl for 10 mins at 37 °C then fixed twice in methanol: acetic acid (3:1). Cells were spotted onto slides, left to air dry then fixed in formalin for 15 mins. Chromosomes were denatured in 2 N HCl with 0.1% Triton X-100 for 15 mins then slides blocked with 10% FBS in PBS/0.1% Triton X-100 for 30 mins. Slides were incubated with anti-BrdU antibody (1:100) in PBS/0.1% Triton X-100 for 2 hours, washed with PBS containing 0.1% Tween-20 then incubated with anti-mouse-FITC antibody (1:200) for 1 hour in the dark. After washing, slides were incubated in 0.01 μg/ml propidium iodide for 1 min, washed again then coverslip mounted with Mounting Media (Vector Laboratories). Metaphases were analyzed using a Zeiss Confocal LSM 780 microscope (Carl Zeiss Pty Ltd).

### Statistics

GraphPad Prism 5 was used to perform two-way ANOVA analyses with Bonferroni post-tests, to assess the significance of differences in clonogenicity and mutagenicity between control and genetically or pharmacologically manipulated cells, exposed to each treatment. In Supplementary Fig. 1a,b, one-way ANOVAs with Dunnett’s multiple comparison tests were used.

## Electronic supplementary material

Supplementary data and title page

## Data Availability

All data generated or analyzed during this study are included in this published article (and its Supplementary Information files).
