# Supplementary material for: Mutagenic assessment of chemotherapy and Smac mimetic drugs in cells with defective DNA damage response pathways
Source: Sci Rep. 2018 Sep 26;8:14421. doi: 10.1038/s41598-018-32517-9 (PMC6158240; doi:10.1038/s41598-018-32517-9)
Supplement: Supplementary file 1 — Supplementary data and title page [file 41598_2018_32517_MOESM1_ESM.pdf]

SUPPLEMENTARY DATA

Mutagenic assessment of chemotherapy and Smac mimetic drugs in cells with defective DNA damage response pathways

Mark A. Miles and Christine J. Hawkins

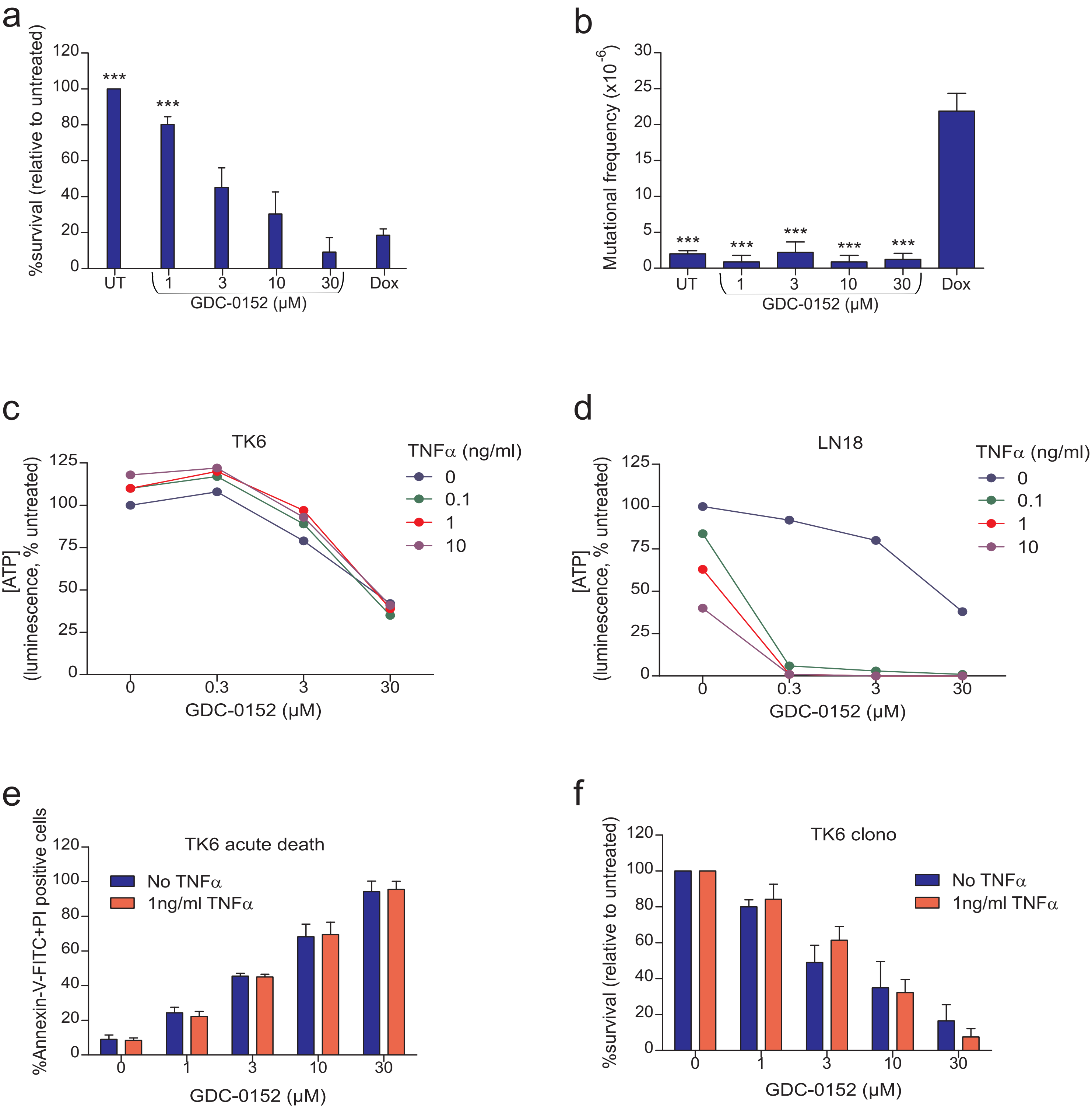

Supplementary Figure 1 - GDC-0152 provokes TNFα-independent death and is non-mutagenic (a) TK6 cells were treated with increasing dose of GDC-0152 or 5 nM doxorubicin for 24 hours. The cells were then processed for clonogenic survival assays. (b) Surviving cells were selected in 6TG to identify emergence of any HPRT mutants. (a, b) One-way ANOVA analyses with Dunnett's multiple comparison test were used to estimate the probability that random chance accounted for the differences observed in responses to doxorubicin versus the various concentrations of GDC-0152. P values < 0.05 are denoted with asterisks: *** < 0.001. (c) TK6 or LN18 (d) cells were incubated for 48 hours with the listed concentrations of GDC-0152 and/or TNFα, or neither. ATP levels were measured with the CellTitreGlo reagent. TK6 cells were also incubated for 24 hours with increasing dose of GDC-0152 in combination with or without 1 ng/ml TNFα and (e) stained for the proportion of cells staining positive for annexin-V and PI or (f) clonogenic survival determined. Error bars represent mean ± SEM from three independent experiments. (e, f) Two-way ANOVA analyses with Bonferroni post-tests were used to estimate the probability that random chance accounted for the differences observed in responses of cells treated in media containing or lacking TNFα. All P values were > 0.5.

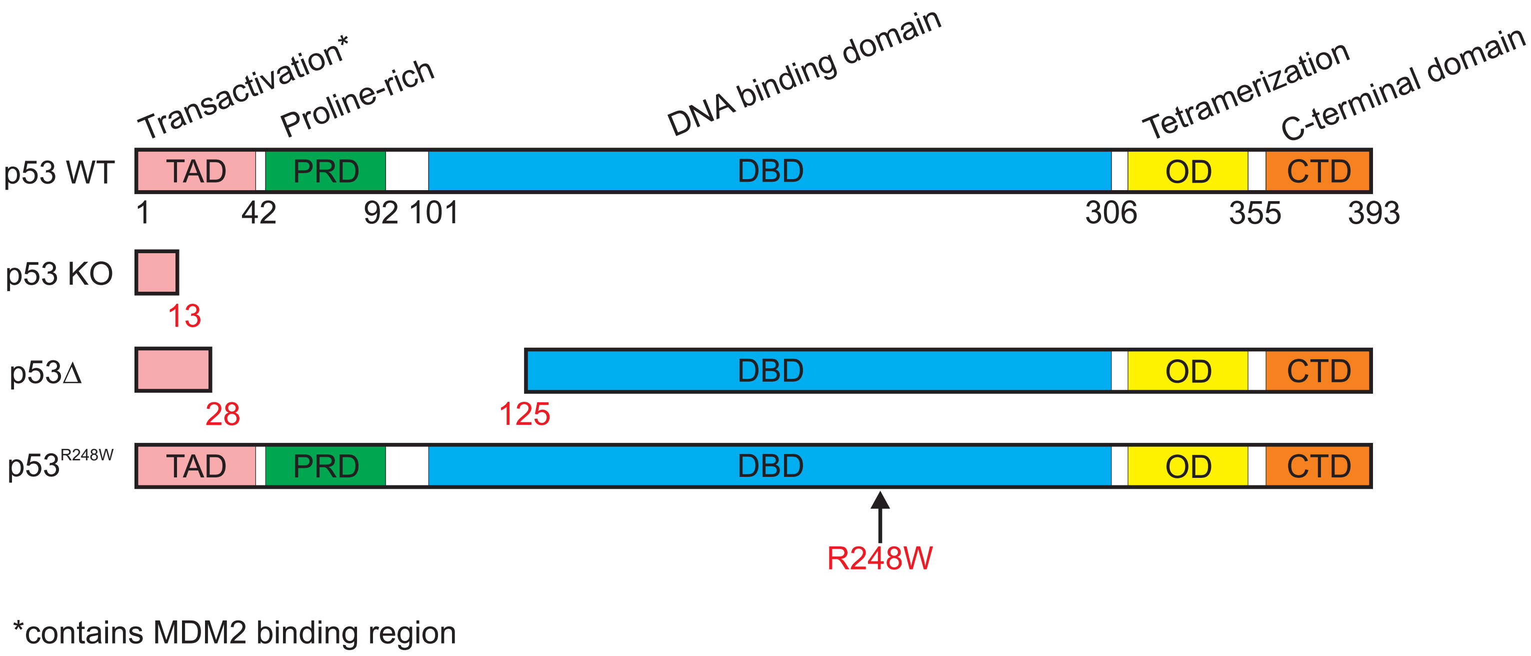

Supplementary Figure 2 - Characterization of p53 CRISPR and R248W mutant clones
Schematic of the wild type p53 protein including key domains and residues, and mutants used in this study. Thick black boxes indicate the predicted translated proteins.

a

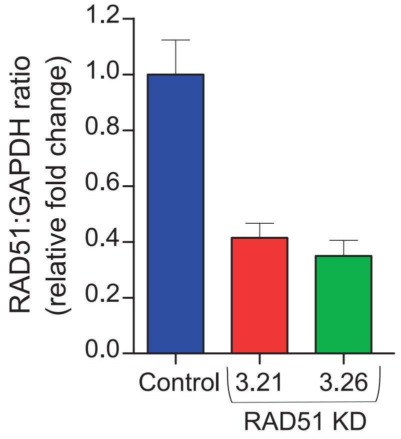

b

Control AGGCTGTTGCCTATGCGCCAAAGAAGGAGCTAATAAATATTAAG
3.21* AGGCTGTTGCCTAT~~GCGCCAAA~~GAAGGAGCTAATAAATATTAAG
3.26* AGGCTGTTGCCTATG~~CGCCAAAG~~AAGGAGCTAATAAATATTAAG
*Heterozygous deletions

c

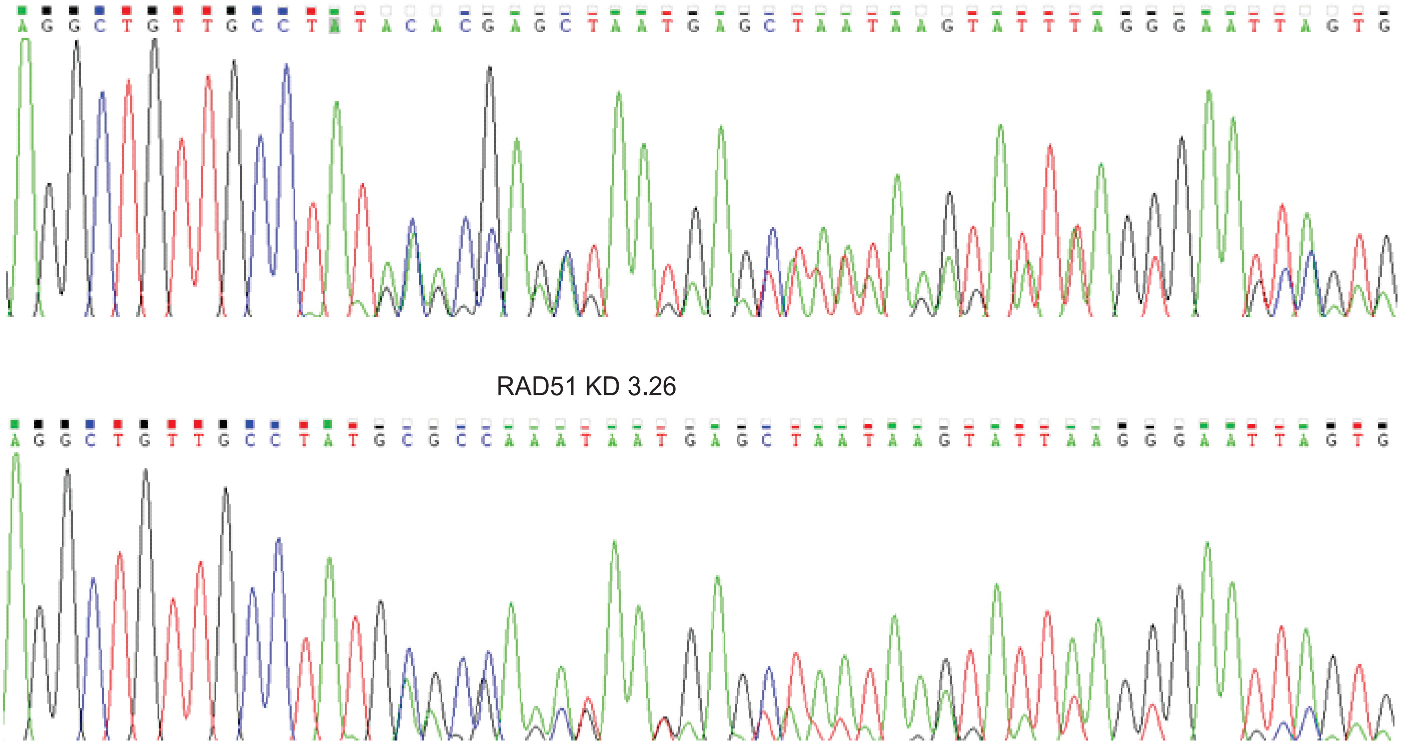

Supplementary Figure 3 - Characterization of RAD51 CRISPR knock-down clones
(a) The intensity of anti-RAD51 bands was quantified using ImageJ software and compared with anti-GAPDH loading control. Error bars represent mean ± SEM from three independent immunoblots. (b) Genomic DNA was extracted from control and RAD51 KD lines and PCR conducted using primers that flank the RAD51 gRNA target region (red). Mutated alleles were identified by sequencing and distinguishing sequences from the two templates that appeared. (c) Chromatogram images indicate the divergence of templates within the gRNA region.

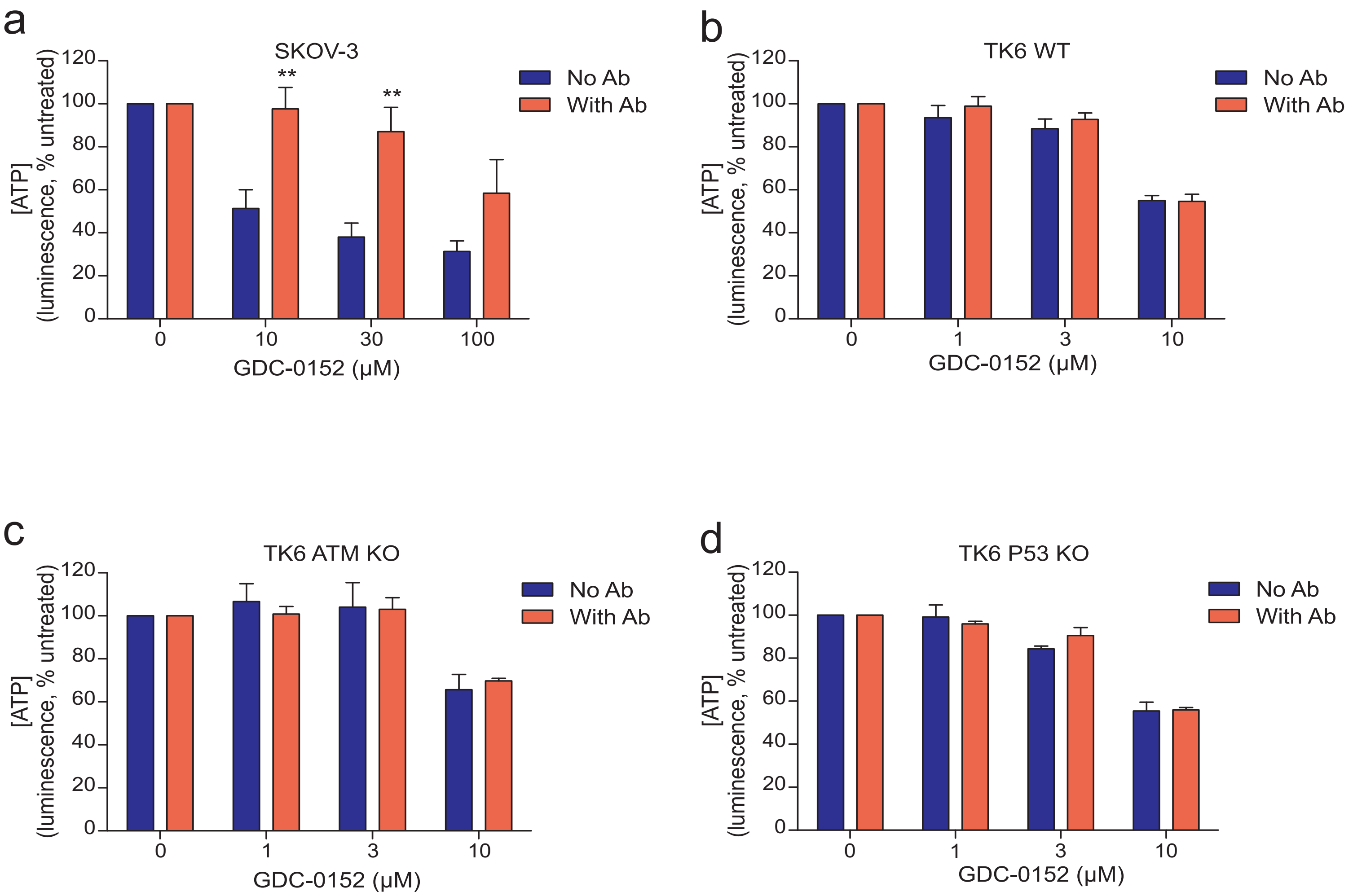

Supplementary Figure 4 - Addition of TNFα neutralizing antibody does not change the sensitivity of TK6 cells to GDC-0152 killing

(a) SKOV-3, (b) TK6 wild type (WT), (c) TK6 ATM KO 1.2, or (d) TK6 p53 KO cells were incubated for 48 hours in media alone or media containing GDC-0152 with or without the addition of 10 ng/ml anti-TNFα antagonistic antibody (Ab). ATP levels were measured with the CellTitreGlo reagent. Error bars represent mean ± SEM from three independent experiments. Two-way ANOVA analyses with Bonferroni post-tests were used to estimate the probability that random chance accounted for the differences observed in responses to each dose between cells treated in media containing or lacking the antibody. P values are designated with asterisks: ** < 0.01. Columns lacking asterisks show data where P values comparing responses of antibody-treated versus untreated cells were > 0.05.

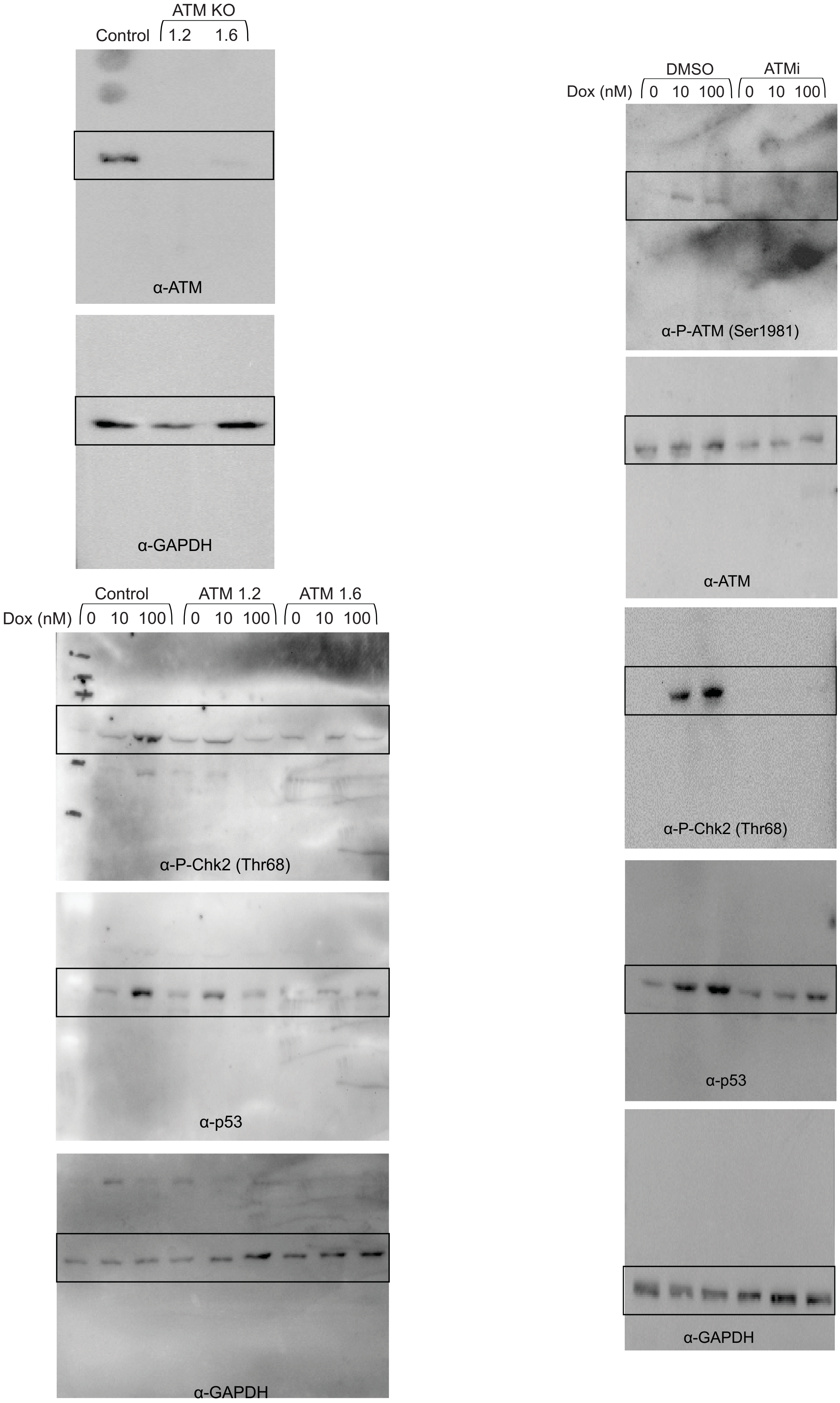

Supplementary Figure 5

Full images of blots in figures 1A, 1B and 1E

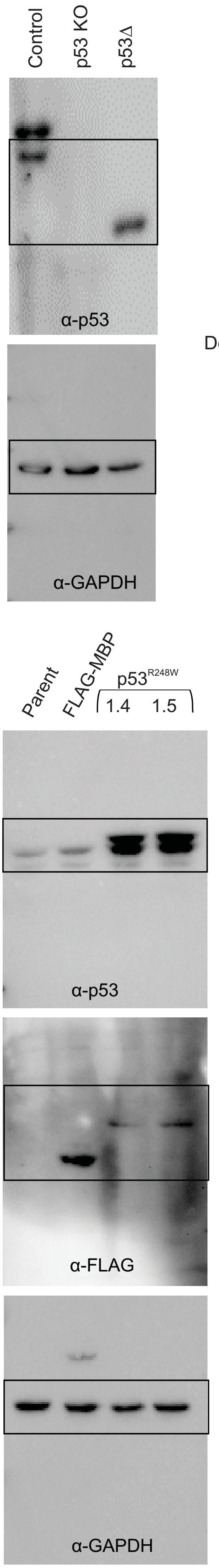

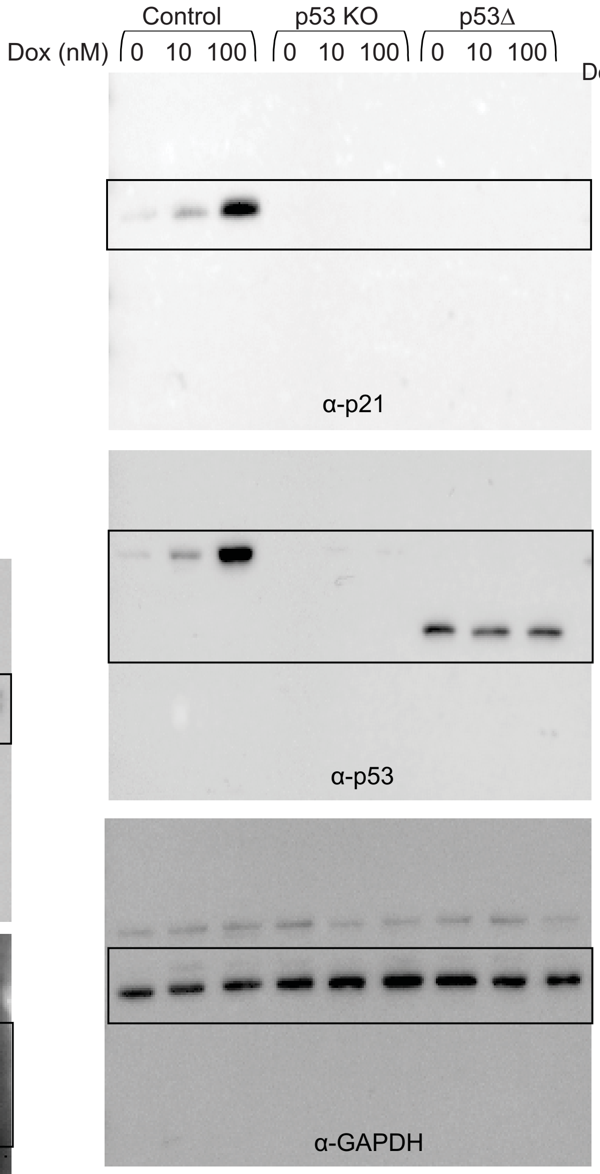

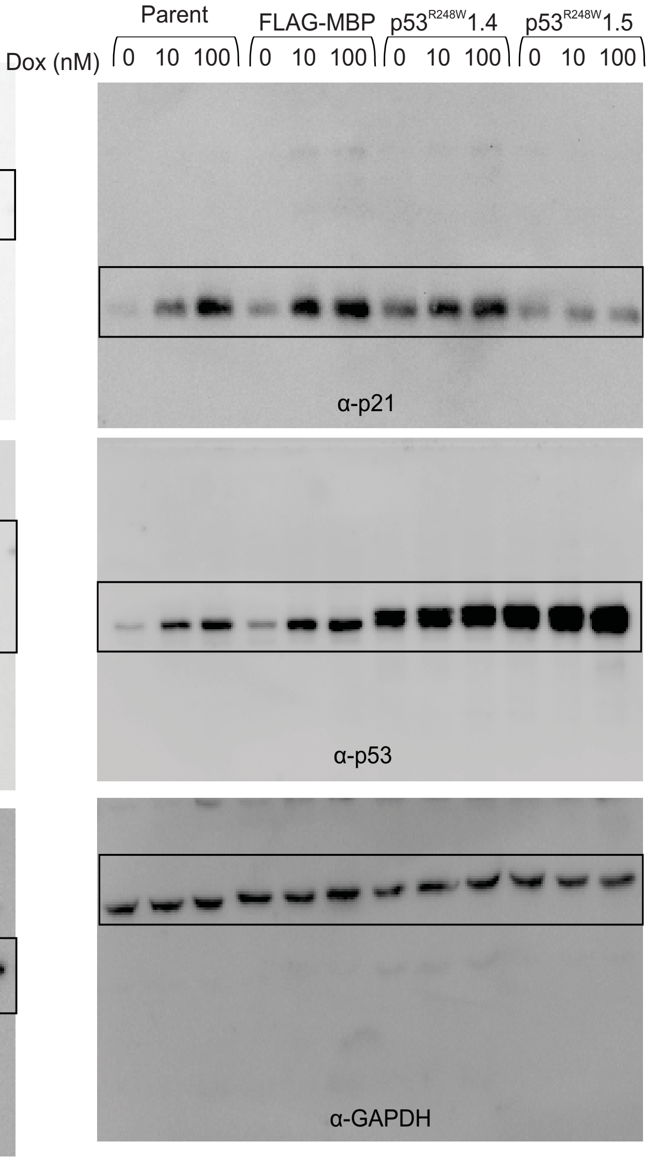

Supplementary Figure 6

Full images of blots in figures 2A, 2B, 2E and 2F

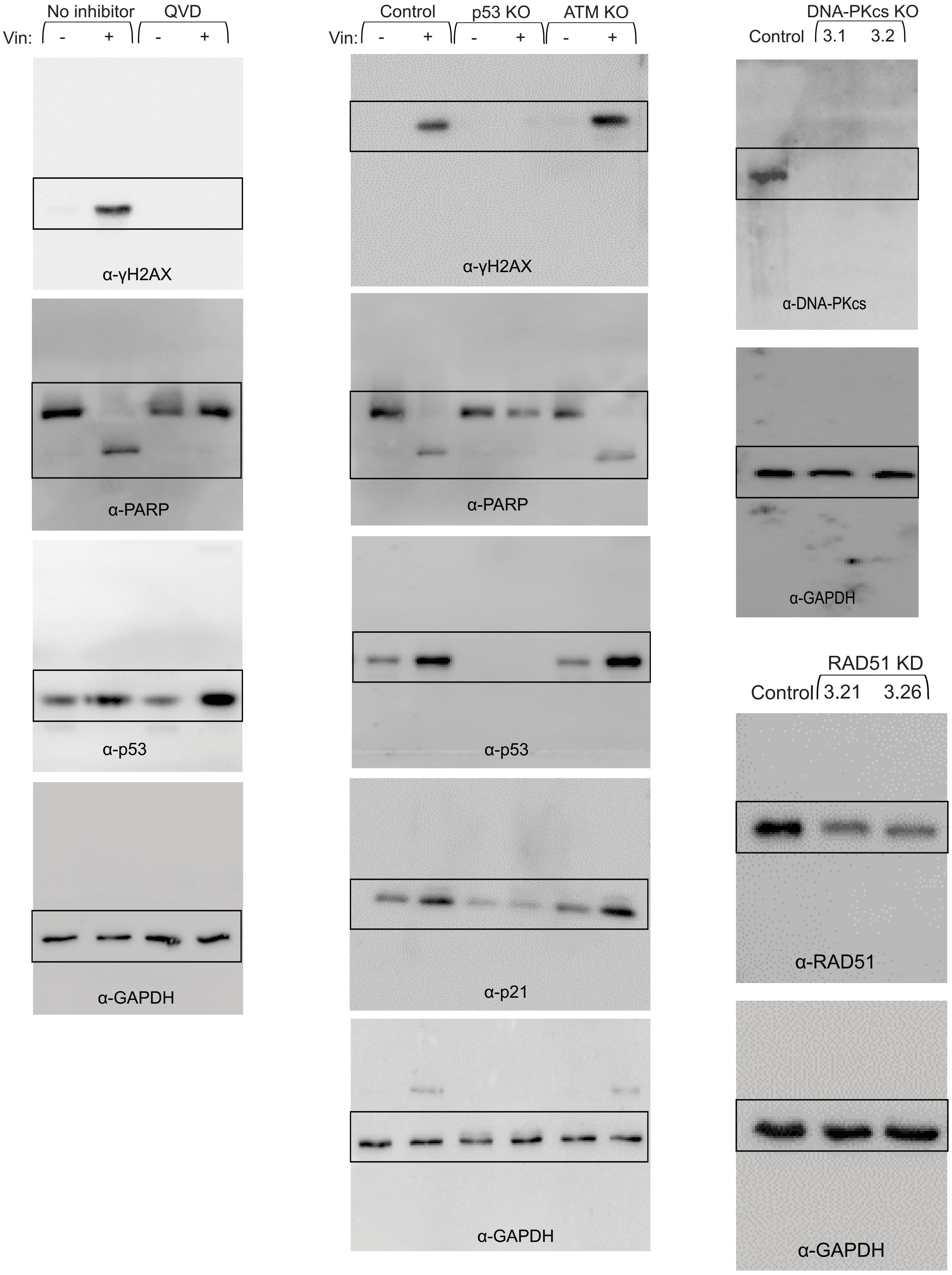

Supplementary Figure 7
Full images of blots in figures 3A, 3B, 4A and 5A
